# Diabetes, Disability and Mortality in Very Old Mexican Americans

**DOI:** 10.1093/geroni/igaa057.3373

**Published:** 2020-12-16

**Authors:** Caroline Kelly, Kyriakos S Markides, Phillip Cantu

**Affiliations:** The University of Texas Medical Branch at Galveston, Galveston, Texas, United States

## Abstract

This study examines the relationship between diabetes, disability and mortality for very old Mexican American, a rapidly growing and understudied segment of the population. Data comes from Wave 7 of the Hispanic EPESE which surveyed 1078 Mexican Americans ≥80 years old, living in the Southwestern United States. Measures included self-reported physician diagnosis diabetes, ADL disability, demographic characteristics, and other self-reported comorbidities. Severe diabetes was defined as being diagnosed more than 10 years ago and/or taking insulin. Diabetes and mortality were studied over a five-year follow-up. Participants with diabetes are more likely to be disabled, 55% prevalence compared to 46% in non-diabetics. Even when controlling for demographic characteristics and comorbidities, the predicted probability of ADL disability in diabetics is 54% compared to 45% in non-diabetics. Logistic regression analysis showed an odds ratio (OR) of 1.51, 95% confidence interval [CI], 1.14-2.02 of any ADL disability among diabetics, controlling for demographic characteristics and comorbidities. Stroke and arthritis were significantly associated with ADL disability as well, OR 2.59, [1.6-4.2] and 1.53 [1.15-2.04] respectively. Diabetics had greater odds of mortality, OR 1.25, than non-diabetics, and among diabetics, severity was predictive of mortality OR 1.38. Gender was an insignificant predictor of mortality. Diabetes prevalence in the very old Mexican American population is increasing and is associated with increased disability and mortality. Increased awareness of the prevalence of diabetes in this population and thus the increased potential of disability is needed to decrease the burden of disease and increase quality of life.

